# Small-Pox

**Published:** 1883-02-20

**Authors:** 


SMALL-POX.—The Small-pox excitement has subsided in Atlanta, no new cases having occurred for some days. The efficacy of vaccination is again vindicated, the thorough vaccination instituted by our city authorities during the present year and the last leaving no subject whom the disease can affect.

